# Transcriptomics Reveals the *ERF2*-*bHLH2*-*CML5* Module Responses to H_2_S and ROS in Postharvest Calcium Deficiency Apples

**DOI:** 10.3390/ijms222313013

**Published:** 2021-12-01

**Authors:** Hong-Ye Sun, Wei-Wei Zhang, Hai-Yong Qu, Sha-Sha Gou, Li-Xia Li, Hui-Hui Song, Hong-Qiang Yang, Wan-Jie Li, Hua Zhang, Kang-Di Hu, Gai-Fang Yao

**Affiliations:** 1 School of Food and Biological Engineering, Hefei University of Technology, Hefei 230009, China; 2018111257@mail.hfut.edu.cn (H.-Y.S.); 2020111400@mail.hfut.edu.cn (S.-S.G.); 2019111426@mail.hfut.edu.cn (L.-X.L.); 2019111437@mail.hfut.edu.cn (H.-H.S.); hzhanglab@hfut.edu.cn (H.Z.); 2 State Key Laboratory of Crop Biology, College of Horticulture Science and Engineering, Shandong Agricultural University, Tai’an 271001, China; zhangww@sdau.edu.cn (W.-W.Z.); hqyang@sdau.edu.cn (H.-Q.Y.); 3 College of Horticulture, Qingdao Agricultural University, Qingdao 266109, China; haiyongqu@qau.edu.cn; 4 Key Laboratory of Cell Proliferation and Regulation Biology, College of Life Science, Beijing Normal University, Ministry of Education, Beijing 100875, China; lwj@bnu.edu.cn

**Keywords:** calcium deficiency, endogenous H_2_S, reactive oxygen species, *ERF2*-*bHLH2*-*CML5* module, postharvest storage quality

## Abstract

Calcium deficiency usually causes accelerated quality deterioration in postharvest fruit, whereas the underlining mechanism is still unclear. Here, we report that calcium deficiency induced the development of bitter pit on the surface of apple peels compared with the healthy appearance in control apples during postharvest storage. Physiological analysis indicates that calcium-deficient peels contained higher levels of superoxide anion (O_2_^•−^), malondialdehyde (MDA), total phenol, flavonoid contents and polyphenol oxidase (PPO) activity, and reduced calcium, H_2_S production, anthocyanin, soluble protein content, and peroxidase (POD) activity compared with those in calcium-sufficient peels. The principal component analysis (PCA) results show that calcium content, ROS, and H_2_S production were the main factors between calcium-deficient and calcium-sufficient apple peels. Transcriptome data indicated that four calmodulin-like proteins (CMLs), seven AP2/ERFs, and three bHLHs transcripts were significantly differentially expressed in calcium-deficient apple peels. RT-qPCR and correlation analyses further revealed that *CML5* expression was significantly positively correlated with the expression of *ERF2/17*, *bHLH2,* and H_2_S production related genes. In addition, transcriptional co-activation of *CML5* by *ERF2* and *bHLH2* was demonstrated by apple transient expression assays and dual-luciferase reporter system experiments. Therefore, these findings provide a basis for studying the molecular mechanism of postharvest quality decline in calcium-deficient apples and the potential interaction between Ca^2+^ and endogenous H_2_S.

## 1. Introduction

Calcium (Ca) is one of the essential and abundant elements for the growth of plants. It not only determines the yield and quality of agricultural crops, but also plays a pivotal role in maintaining plant cell structure, resistance to adverse stress and signal transduction [1,2,3,4]. Calcium can mitigate stress conditions, for example, by neutralizing reactive oxygen species (ROS) produced in cells [5,6,7]. Calcium deficiency results in ROS accumulation and causes damage to membranes, reduces the cell wall, weakens tissue stiffness, and increases water loss, which in turn leads to leaf wilting and shortens the shelf life of harvested fruit [8,9,10]. It also causes various physiological disorders, such as reduction in fruit size, low firmness and thin peel, tip burn, and oxidative stress [5,10,11,12]. It has also been reported that calcium deficiency causes bitter pit and decreases titratable acidity, total soluble solids, and vitamin C contents, resulting in poorer postharvest fruit quality [13]. Bitter pit is one of the major post-harvest disorders associated with apple production and can cause up to a 50% post-harvest loss [14].

Ca^2+^ is one of the most important second messengers in plant cell signaling [15]. Ca^2+^ signaling regulates numerous abiotic stress reactions [16]. Calmodulin (CaM) and calmodulin-like (CML) proteins [17] are conventional Ca^2+^-binding proteins, which are generally comprised of one to six EF-hand motifs [18]. It has been reported that CMLs initiate cellular responses by binding to relevant transcription factors (TFs) (e.g., AP2/ERFs and bHLHs) and transmitting calcium signaling downstream [19]. It is reported that *SlCML44* has a critical effect on Ca^2+^ signaling during abiotic stress tolerance in tomato fruit [20]. In rice, *OsERF48*-OE induces modulation of *OsCML16*, thereby enhancing drought tolerance and root growth [21]. Nevertheless, whether and how ERFs/bHLHs regulate CMLs via calcium signaling in the postharvest fruit suffering from bitter pit due to calcium deficiency is still largely unknown. It has been reported that Ca^2+^ interacts with H_2_S and plays a vital role in plant growth and development by regulating a range of physiological processes and imparting abiotic stress tolerance [22]. For example, the application of exogenous NaHS increases the intracellular Ca^2+^ content under both hypoxia and heat stress in tobacco [23,24]. However, there were few reports on the mode of interaction between Ca^2+^ and H_2_S.

Hydrogen sulfide (H_2_S) is an important signal molecule [25], participating in the regulation of plant development, and resistance to stress conditions [26]. In plants, the cysteine desulfhydrases (CDs) are responsible for the majority of endogenous H_2_S production and L/D-cysteine desulfhydrase (LCD/DCD) catalyze the production of H_2_S with L-cysteine and D-cysteine as the substrates, respectively [27]. Moreover, H_2_S can also be produced by the O-acetyl-L-serine (thiol) lyase (OASTL) family proteins and sulfite reductase (SiR) [28]. A number of studies have reported that H_2_S delayed postharvest senescence of various fruits and vegetables [29]. For example, postharvest treatment of H_2_S was found to delay the softening of tomato [30] and Chilean strawberry (*Fragaria chiloensis*) fruit [31], thereby slowing postharvest senescence. In addition, H_2_S can help to eliminate excessive ROS in harvested kiwifruit by activating antioxidative systems [32]. A previous report suggested a possible association between ROS and H_2_S, and calcium homeostasis in wheat coleoptiles (*Triticum aestivum* L.) largely dictates the formation of ROS [33]. Thus, we propose a hypothesis that calcium deficiency may affect the production of endogenous H_2_S and increase ROS, thereby reducing fruit quality during postharvest storage. However, the molecular mechanism of Ca^2+^ on the production of endogenous H_2_S has rarely been reported, and the role of endogenous H_2_S in postharvest quality deterioration are still unclear.

Apple (*Malus domestica*), a Rosaceae fruit, is of important economic value. Apple contains multiple beneficial and healthy components, which include vitamins and anthocyanins. Due to the antioxidant and anti-inflammatory properties, apples are often employed to maintain a well-balanced diet [34]. Mineral nutrients, particularly calcium, are critical to the growth of plants, fruit quality, and productivity [35]. Previous study has reported that calcium deficiency causes the formation of bitter pit on the surface of apple peels [14], which affects the storage quality and storage life of apples. Postharvest calcium treatments have been shown to be effective in reducing physiological diseases in apples, delaying aging, and greatly maintaining the quality of the fruit [36,37]. However, whether calcium deficiency affects the production of endogenous H_2_S and the interaction between calcium, H_2_S, and ROS are largely unknown in calcium deficient apples during postharvest storage.

In this study, the effects of calcium deficiency on ROS and H_2_S production in apples were elucidated by the physiological parameters, principal component and correlation analysis. Then, differentially expressed calcium-regulated genes were screened from transcriptome data, and their expression was verified by RT-qPCR. Moreover, the expression of genes related to H_2_S production was also analyzed to reveal the effect of calcium deficiency on H_2_S production. Further, *ERF2*-*bHLH2* complex was found to activate the expression of *CML5* by apple transient expression assays and dual-luciferase reporter system experiments, thereby enhancing the understanding of the regulatory mechanism of calcium-deficient apple peels and the potential interaction between Ca^2+^ and endogenous H_2_S, contributing to improving the appearance quality of apple fruit. 

## 2. Results

### 2.1. Calcium Deficiency Significantly Affects the Phenotype of Apple Peels and Reduces H_2_S Production

**Figure 1:**
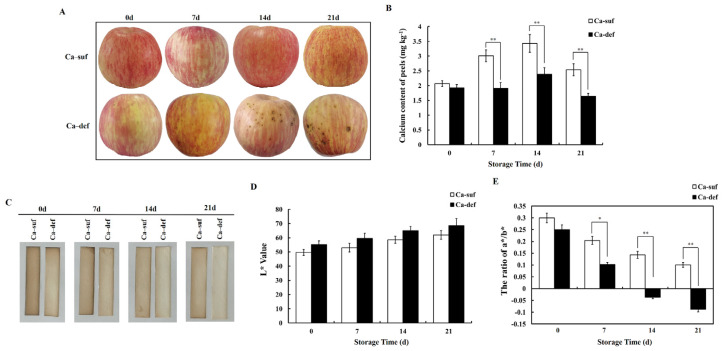
Comparison of phenotype and endogenous H_2_S determination of apple peel at 0, 7, 14, and 21 DAS with calcium deficiency and calcium sufficiency. (**A**) Phenotypic change of apple peel at 0, 7, 14, and 21 DAS. Ca-suf, Ca sufficiency; Ca-def, Ca deficiency. Data are presented as means ± SD (n = 5). (**B**) Calcium contents of calcium-sufficient and calcium-deficient apple peels during storage. Data are presented as means ± SD (n = 3). (**C**) H_2_S production capacity from calcium-sufficient and calcium-deficient apple peels during storage as detected by the brown precipitate, lead sulfide. Data are presented as means ± SD (n = 6). (**D**,**E**) represent the change of color-parameter L* value and the ratio of a*/b* corresponding to (**C**). L* represents luminance; a* represents a range from green to magenta; b* represents a range from yellow to blue. Data are presented as means ± SD (n = 6). Asterisks indicate statistical difference of the values at *p* < 0.05 (*); *p* < 0.01 (**).

In this experiment, the phenotypes of calcium-deficient and calcium-sufficient apple fruits stored for different days after harvest were observed. As shown in Figure 1A, the apple fruit did not significantly differ in size, but the color and smoothness of apple peels were significantly different at 0, 7, 14, and 21 days after storage (DAS) between calcium-deficient and calcium-sufficient apples. The appearance of calcium-sufficient apple peels showed no obvious change with increasing days of storage. At 7 DAS, however, bitter pit appeared on calcium-deficient apple peels and increased continuously with increasing days of storage. At the same time, the peel color of calcium-deficient apples gradually deteriorated, and the surface became rough. Then, the calcium contents in calcium-deficient and calcium-sufficient apple peels at different storage times were measured. The calcium content of calcium-deficient apple peels was always lower than that of calcium-sufficient apple peels during storage, but significant difference between the two was observed at 7, 14, and 21 DAS (*p* < 0.01) (Figure 1B). It is evident that calcium deficiency in apples affects the phenotype of apple peels and leads to physiological disorders. Hence, it is important to investigate the molecular mechanism of calcium deficiency-induced disorders in apples.

In order to measure H_2_S directly, lead sulfide method was applied to show the changes of H_2_S production in calcium-deficient and sufficient apple peels. As shown in Figure 1C, more H_2_S was produced by calcium-sufficient apple peels than by calcium-deficient apple peels at 0, 7, 14, 21 DAS, evidenced by the darkened precipitate due to lead sulfide formed on the strips. Luminance (L*) and color change (a*/b*) was measured by colorimeter, and they could show the effect of calcium deficiency on H_2_S production. Higher L* values and lower ratio of a*/b* indicated that less H_2_S was absorbed by the lead acetate filter paper. The L* values were higher for calcium-deficient apple peels than for calcium-sufficient apple peels (Figure 1D), but the ratio of a*/b* was reversed (Figure 1E). In summary, it is clear that calcium deficiency reduces H_2_S production in apple peels.

### 2.2. Calcium Deficiency Increases the Contents of Flavonoids and Total Phenols but Decreases Anthocyanin Contents in Apple Peels

**Figure 2:**
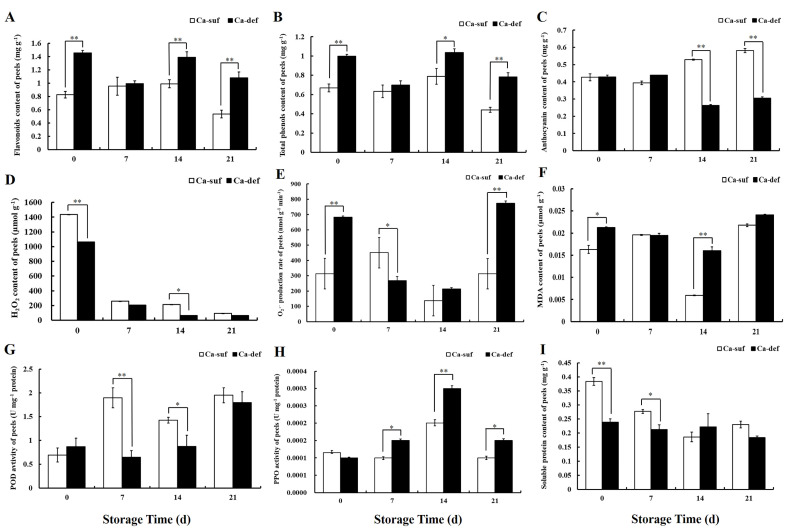
Effects of Ca-suf and Ca-def on the contents of (**A**) flavonoids, (**B**) total phenols, (**C**) anthocyanin, (**D**) hydrogen peroxide (H_2_O_2_), (**E**) superoxide anion (O_2_^•−^), and (**F**) malonaldehyde (MDA) as well as on (**G**) peroxidase (POD) activity, (**H**) polyphenol oxidase (PPO) activity and (**I**) soluble protein in apple peels at 0, 7, 14, and 21 DAS. Ca-suf, Ca sufficiency; Ca-def, Ca deficiency. Data are presented as means ± SD (n = 3). Asterisks indicate statistical difference of the values at *p* < 0.05 (*); *p* < 0.01 (**).

To further explore the mechanism by which calcium deficiency affects the phenotype of apple peels, the contents of flavonoids, total phenols, and anthocyanins were determined. As shown in Figure 2A, the flavonoid contents of calcium-deficient peels were almost indistinguishable from those of calcium-sufficient peels at 7 DAS, whereas at 0, 14, and 21 DAS, the flavonoid contents of calcium-deficient peels were significantly higher than those of calcium-sufficient peels (*p* < 0.01). The total phenol contents of calcium-deficient peels were higher than those of calcium-sufficient peels, and the difference was significant at 0, 14, and 21 DAS (*p* < 0.01 or *p* < 0.05) (Figure 2B). At the same time, there was no appreciable difference in anthocyanin contents between calcium-deficient and calcium-sufficient peels at 0 and 7 DAS, but the anthocyanin contents in calcium-deficient peels were significantly lower than those in calcium-sufficient peels at 14 and 21 DAS (*p* < 0.01) (Figure 2C).

### 2.3. Calcium Deficiency Induces the Production of ROS in Apple Peels

Excessive production of ROS and oxidative damage are usually observed during fruit storage. To investigate the effect of calcium deficiency on ROS production in apple peels, the accumulation of O_2_^•−^, H_2_O_2_, and MDA was determined. Compared to calcium-sufficient peels, the H_2_O_2_ contents of calcium-deficient peels were lower during postharvest storage days; in particular, the most remarkable variation was at 0 DAS (*p* < 0.01) (Figure 2D). The O_2_^•−^ production rate was significantly higher in calcium-deficient peels than in calcium-sufficient peels at 0 and 21 DAS (*p* < 0.01), but the difference was not significant at 14 DAS (Figure 2E). Similarly, the MDA contents in calcium-sufficient peels were lower than those in calcium-deficient peels, especially at 14 DAS (*p* < 0.01) (Figure 2F).

### 2.4. Changes in POD Activity, PPO Activity Soluble Protein Content between Calcium Deficiency and Sufficiency Conditions

To study the potential mechanism of calcium deficiency on the accumulation of ROS, the peroxidase (POD) and polyphenol oxidase (PPO) activities were determined in calcium deficiency and sufficiency apples. As shown in Figure 2G, the POD activity in calcium-sufficient peels was maintained at a higher level than that in calcium-deficient peels at 7 and 14 DAS (*p* < 0.05 or *p* < 0.01). The POD activity gradually increased with increasing days of storage and then decreased. The PPO activity in calcium-deficient peels was significantly higher than that in calcium-sufficient peels at 14 DAS (*p* < 0.01) (Figure 2H). To compare the effects of calcium deficiency on apple peel quality, soluble protein content was measured. The soluble protein content sustained at a lower level in calcium-deficient peels than that in the calcium-sufficient peels at 0, 7, 14, and 21 DAS (Figure 2I).

### 2.5. PCA Analysis of the Bioactive Substance Changes in Apple Peels

**Figure 3:**
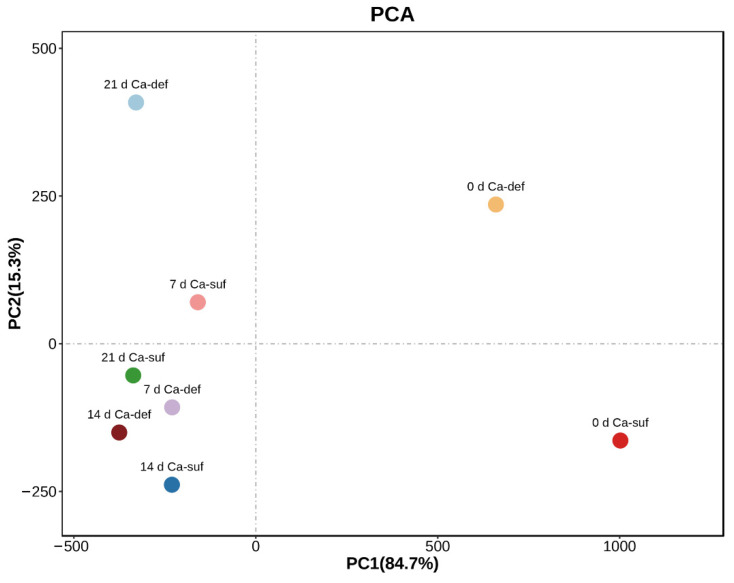
PCA of the main metabolites in apple peels during storage periods. PC1 and PC2, respectively, represented the contribution rate of principal components. Ca-suf, Ca sufficiency; Ca-def, Ca deficiency.

The principal component analysis (PCA), a statistical method of dimensionality reduction, was carried out to investigate the main factors of the bioactive substances in apple peels that were most affected by calcium deficiency, and O_2_^•−^ production rate, ratio of a*/b*, L* value, POD and PPO activity, H_2_O_2_, calcium, soluble protein, flavonoid, total phenolics, anthocyanins, MDA contents were used in PCA (Figure 3). The contribution rates of PC1 and PC2 were 84.7% and 15.3%, respectively. There was a significant difference between calcium-deficient apple peels and calcium-sufficient apple peels. In PC1, H_2_O_2_, calcium content, O_2_^•−^ production rate, and ratio of a*/b* were the main factors, while POD activity, L* value, and O_2_^•−^ production rate were the main factors in PC2 (Table 1). The above results indicated that calcium deficiency caused physiological disorders and changes in postharvest storage quality in apple peels, which severely affected the ROS, H_2_S production, and redox processes in apple peels.

### 2.6. Identification and GO Classification and KEGG Enrichment Analysis of DEGs between Calcium-Deficient and Calcium-Sufficient Apple Peels

To investigate the molecular mechanisms of calcium regulation in apple peels, differentially expressed genes (DEGs) associated with calcium regulation between calcium-sufficient and calcium-deficient apple peels were obtained from published transcriptome data. Pairwise comparison of the three sequenced samples resulted in three sets of contrasts with T02/T01, T03/T01, and T03/T02 (T01, diseased peel of calcium-deficient apples; T02, healthy peel of calcium-deficient apples; and T03, peel of calcium-sufficient apples). In the T02/T01, T03/T01, and T03/T02 comparisons, 1323, 2880, and 2182 DEGs were upregulated, respectively, while 1573, 1606, and 269 DEGs were downregulated, respectively (Figure S1A–C). In total, 1031 DEGs were common to all three comparisons (Figure S1D).

GO classification analysis was used to investigate the gene expression profiles of calcium-deficient apple peels, and the T03/T01 comparison with the highest number of DEGs was selected for gene annotation analysis. The DEGs were classified into 51 functional groups based on their biological processes (Figure S1E). The major subcategories were as follows: 20 subcategories for biological process, 15 subcategories for cellular component, and 16 subcategories for molecular function. The DEGs in ‘signaling’ and ‘antioxidant activity’ played important roles during the calcium regulated processes. These results provided a comprehensive perspective for screening candidate genes involved in calcium regulation.

KEGG analysis provided information and further understanding of secondary metabolites induced by calcium signaling. As shown in Figure S1F, 20 pathways were enriched with over 20 DEGs. Plant hormone signal transduction was the most abundant DEG found in metabolism category followed by plant–pathogen interaction, biosynthesis of amino acids, and phenylpropanoid biosynthesis. These results suggested that the DEGs in the T03/T01 comparison were mainly enriched in metabolic processes related to plant signal transduction and plant pathogen infestation.

### 2.7. Analysis of Transcriptomics on Calcium-Deficient Peels and Gene Expression Validation

**Figure 4:**
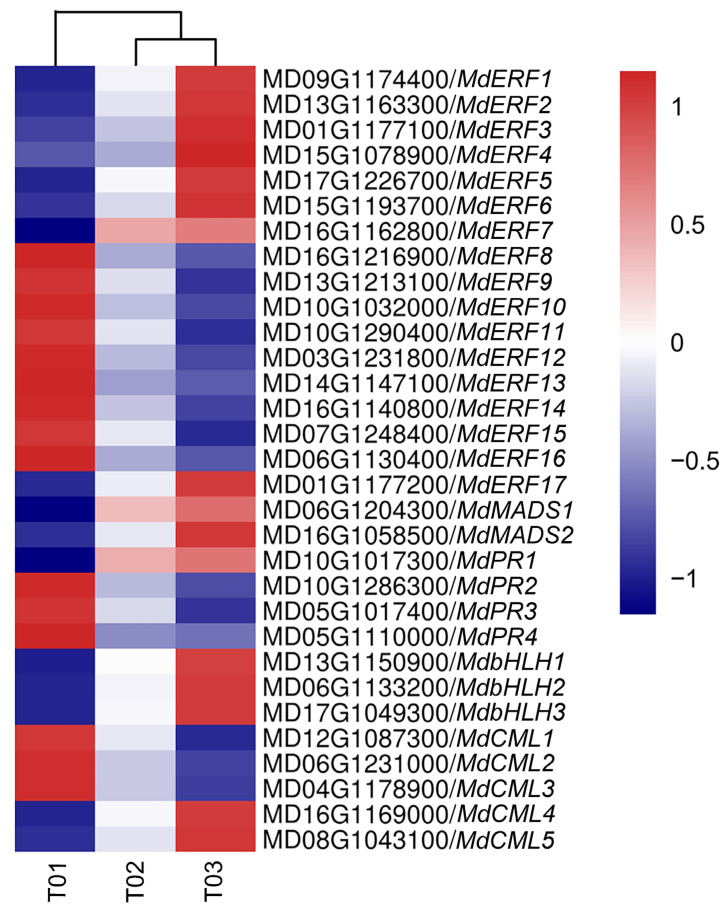
Heatmap of selected calcium related genes and co-expressed transcription factors. T01, diseased peels of calcium-deficient apples; T02, healthy peels of calcium-deficient apples; T03, peels of calcium-sufficient apples. The color of the scale label from red to blue represents the change in RPKM value from ‘1’ to ‘−1’ by Z-score normalization.

In order to explore the molecular mechanisms of calcium regulation in apple peels, calmodulin-like proteins (CMLs), pathogen-related proteins (PRs), and several costimulatory TFs, including AP2/ERFs, bHLHs, and MADS-box, were screened from the 1031 DEGs co-expressed in the three sets of sequenced samples. Heatmap analysis was performed on 5 CMLs, 4 PRs, 17 AP2/ERFs, 3 bHLHs, and 2 MADSs (Figure 4). These candidate genes were differentially expressed in apple peels due to calcium deficiency with two expression patterns. Among them, *CML1-3*, *PR2-4,* and *ERF8-16* were upregulated, and *CML4*, *CML5*, *PR1*, *ERF1-7*, *ERF17*, *bHLH1-3*, *MADS1,* and *MADS2* were downregulated. *CML1-3*, *CML5*, *PR1-4*, *ERF2*, *ERF5*, *ERF13-17*, *bHLH1-3*, *MADS1,* and *MADS2* had the most significantly different expression.

**Figure 5:**
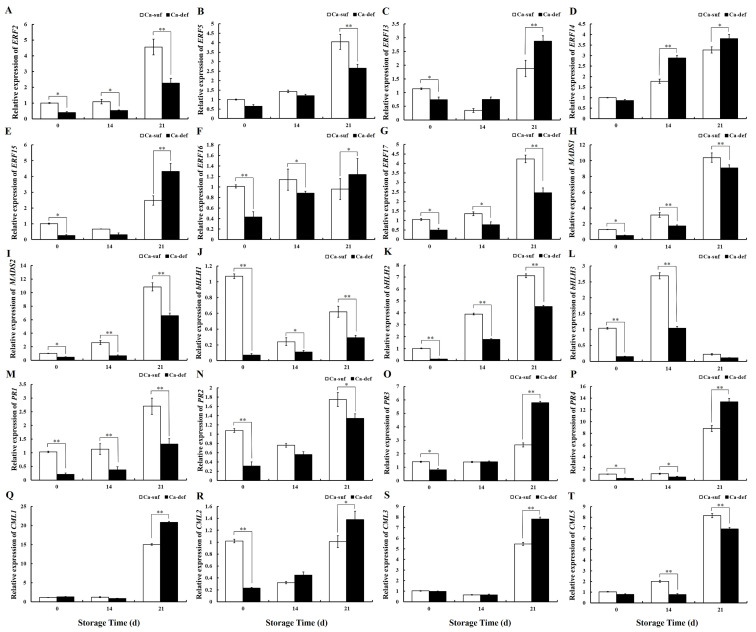
Expression pattern analysis of (**A**) *ERF2,* (**B**) *ERF5,* (**C**) *ERF13,* (**D**) *ERF14,* (**E**) *ERF15,* (**F**) *ERF16,* (**G**) *ERF17,* (**H**) *MADS1,* (**I**) *MADS2,* (**J**) *bHLH1,* (**K**) *bHLH2,* (**L**) *bHLH3,* (**M**) *PR1,* (**N**) *PR2,* (**O**) *PR3,* (**P**) *PR4,* (**Q**) *CML1,* (**R**) *CML2,* (**S**) *CML3,* and (**T**) *CML5* in apple peels under calcium sufficiency and deficiency with storage at 0, 14, and 21 DAS by RT-qPCR. Data are presented as means ± SD (n = 3). Asterisks indicate statistical difference of the values at *p* < 0.05 (*); *p* < 0.01 (**).

As heatmap analysis of DEGs yielded seven AP2/ERFs (*ERF2*, *ERF5*, *ERF13, ERF14, ERF15, ERF16,* and *ERF17*), three bHLHs (*bHLH1, bHLH2,* and *bHLH3*), two MADSs (*MADS1* and *MADS2*), four PRs (*PR1, PR2, PR3,* and *PR4*), and four CMLs (*CML1, CML2, CML3,* and *CML5*) with significant differential expression, real-time qPCR was utilized to further validate their expression patterns in calcium-sufficient and calcium-deficient apple peels (Figure 5 and Figure S2). The expression patterns of *ERF2, ERF5, ERF13, ERF14, ERF17, bHLH1, bHLH2, bHLH3, MADS1, MADS2, PR1, PR3, CML1, CML2, CML3,* and *CML5* were consistent with those obtained by transcriptome sequencing, and the correlation coefficient was 0.7722 (Figure S3). Among them, the expression of *ERF2* and *ERF17* was higher than that of *ERF5*, *ERF13,* and *ERF14*, and *bHLH2* expression level was also higher than that of *bHLH1* and *bHLH3*. *ERF2*/*17*, *bHLH2,* and *CML5* showed consistent expression patterns, and the differences in the expression of them were significant between calcium-deficient and calcium-sufficient peels. 

**Figure 6:**
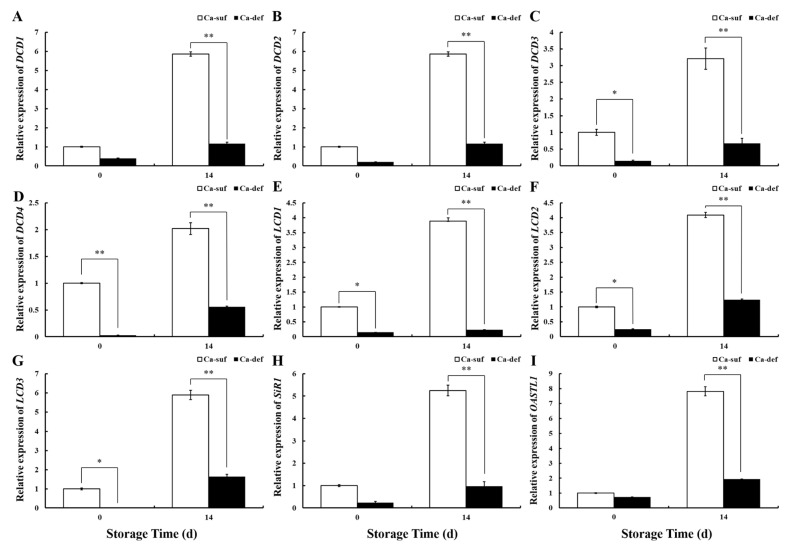
Expression pattern analysis of genes (**A**) *DCD1,* (**B**) *DCD2,* (**C**) *DCD3,* (**D**) *DCD4,* (**E**) *LCD1,* (**F**) *LCD2,* (**G**) *LCD3,* (**H**) *SiR1,* and (**I**) *OASTL1* related to H_2_S production in apple peels under calcium sufficiency and deficiency with storage at 0 and 14 DAS by RT-qPCR. Data are presented as means ± SD (n = 3). Asterisks indicate statistical difference of the values at *p* < 0.05 (*); *p* < 0.01 (**).

For the investigation of the expression of genes related to H_2_S production in apple peels by calcium deficiency, four DCDs (*DCD1, DCD2, DCD3,* and *DCD4*), three LCDs (*LCD1, LCD2,* and *LCD3*), one SiR (*SiR1*), and one OASTL (*OASTL1*) genes were selected for real-time qPCR at 0 and 14 DAS. As shown in Figure 6, whether at 0 or 14 DAS, *DCD1, DCD2, DCD3, DCD4, LCD1, LCD2, LCD3, SiR1,* and *OASTL1* expressions in calcium-deficient apple peels were lower than in calcium-sufficient apple peels, while at 14 DAS, *DCD1, DCD2, DCD3, DCD4, LCD1, LCD2, LCD3, SiR1,* and *OASTL1* expressions in calcium-deficient peels were significantly lower than in calcium-sufficient peels (*p* < 0.01). These results were consistent with decreased H_2_S production in calcium-deficient apple peels, suggesting that calcium deficiency caused a decrease in the production of H_2_S from apple peels by reducing the expression of genes related to H_2_S production.

### 2.8. Correlation Analysis between Genes Expression and Physiological Parameters in Apple Peels 

**Figure 7:**
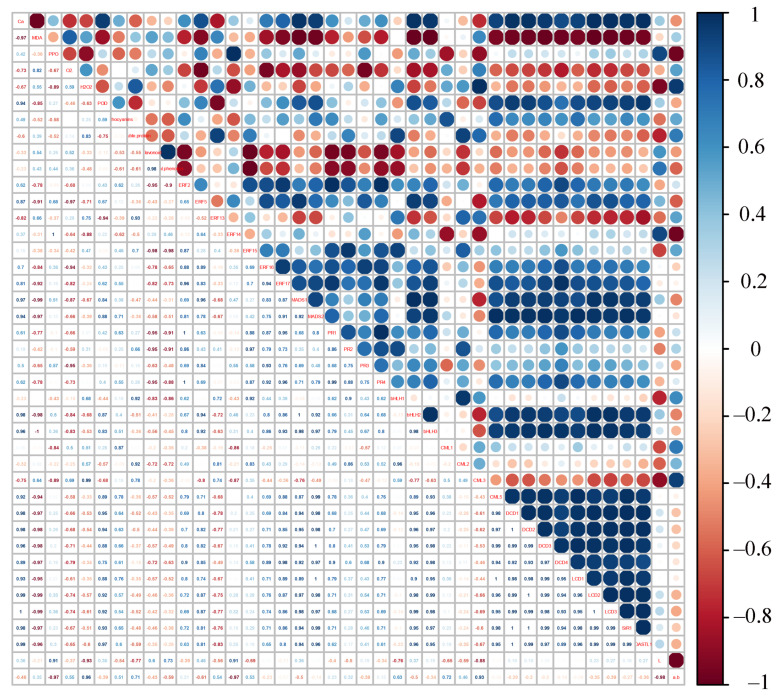
Correlation analysis between gene expression and identified indices in apple peels. R scripts were used to analyze Pearson’s correlation coefficients. The color of the scale label from blue to red represents the change in Pearson’s correlation coefficients from ‘1’ to ‘−1’ with the following indicators: ‘+’ represents a positive correlation, ‘−’ represents a negative correlation, 0.8–1 represents a highly strong correlation, 0.6–0.8 represents a strong correlation, 0.4–0.6 represents a moderate correlation, 0.2–0.4 represents a weak correlation, and 0–0.2 represents a very weak correlation or no correlation.

The result of the correlation analysis between genes expression and physiological parameters in apple peels is shown in Figure 7. Calcium content was negatively correlated with MDA, O_2_^•−^, H_2_O_2_ contents, and ratio of a*/b*, positively correlated with POD activity, H_2_S production on filter papers, *DCD1, DCD2, DCD3, DCD4, LCD1, LCD2, LCD3, SiR1,* and *OASTL1* expression levels, indicating that calcium deficiency activated ROS production in apples but reduced H_2_S production. The expression levels of *ERF2, ERF17, bHLH2,* and *CML5* were positively correlated with calcium content, PPO activity, POD activity, H_2_S production on filter papers, *DCD1, DCD2, DCD3, DCD4, LCD1, LCD2, LCD3, SiR1,* and *OASTL1* expression levels, but negatively correlated with MDA, O_2_^•−^, H_2_O_2_ contents, and ratio of a*/b*. Moreover, H_2_S production showed positive correlation with MDA, O_2_^•−^, H_2_O_2_ contents in apple peels. Based on Figure 7 and the above results, it could be concluded that there was a strong positive correlation between the expression of presumably *ERF2*, *ERF17*, *bHLH2,* and *CML5*, and they were highly expressed and differ significantly between calcium-deficient and calcium-sufficient apple peels at storage time. It was therefore hypothesized that *ERF2, ERF17,* and *bHLH2* may be involved in the regulation of *CML5* and H_2_S production during postharvest apple fruit storage.

### 2.9. ERF2-bHLH2 Coregulation Promotes CML5 Expression in Apple Peels

**Figure 8:**
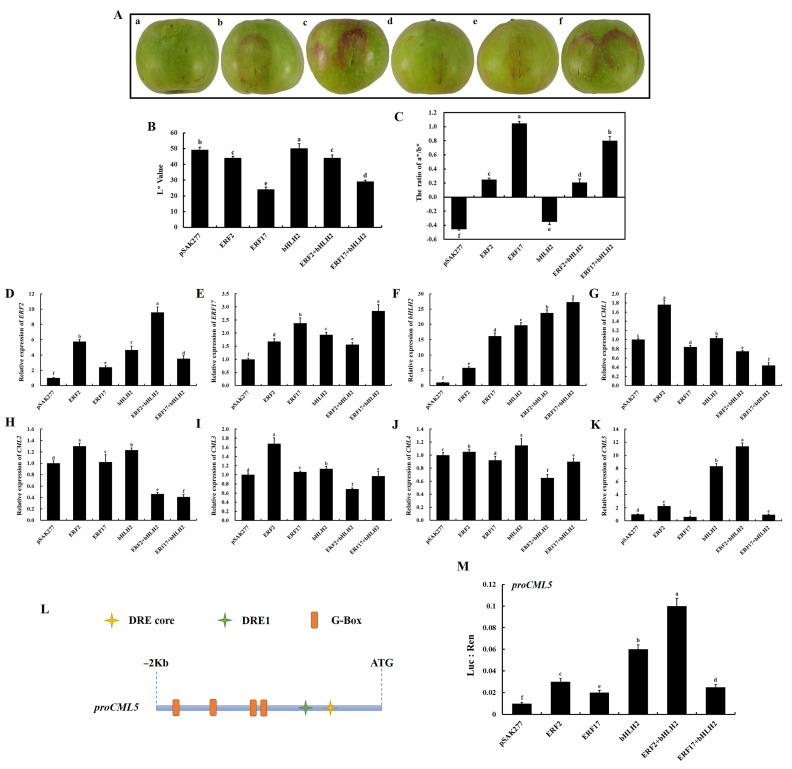
*ERF2*-*bHLH2* coregulation promotes *CML5* expression in apple peels. (**A**) Apple peel phenotypes are shown for the following transient transformations: (**a**), pSAK277; (**b**), *ERF2*; (**c**), *ERF17*; (**d**), *bHLH2*; (**e**), *ERF2* + *bHLH2*; (**f**), *ERF17* + *bHLH2*. Data are presented as means ± SD (n = 5). (**B**,**C**) The L* and a*/b* ratio color parameter values indicate the color changes. The values are presented as the means ± SD (n = 6). (**D**–**K**) Expression levels of *ERF2, ERF17, bHLH2, CML1, CML2, CML3, CML4,* and *CML5* in apple peels from (**A**(**a**–**f**)). The values are presented as the means ± SD (n = 3). (**L**) Predicted cis-acting elements in the upstream 2 kb promoter regions of *CML5, ERF2, ERF17,* and *bHLH2*. Pro, promoter. The DRE1 and DRE core are ERFs cis-acting elements, and the G-Box is bHLHs cis-acting element. (**M**) Validation of the activation effect by cotransformation of *ERF2*/*ERF17* and *bHLH2* on the *CML5* promoter using a dual-luciferase assay in tobacco leaves. The ratio of Luc to Ren indicates that TFs activate the promoter activity of *CML5*. The values are presented as the means ± SD (n = 3). Uppercase letters represent statistical difference at *p* < 0.01, and lowercase letters represent statistical difference at *p* < 0.05.

To further investigate whether and how *ERF2*/*17* and *bHLH2* regulate *CML5* in apple peels, a transient transformation experiment was performed using ‘Honeycrisp’ apples at 60 days after flower blooming, and *ERF2*/*ERF17* and *bHLH2* were individually or cotransformed in apples via *Agroinfiltration*. Transient expression assays in apples showed that individual transformation of *ERF2* and *bHLH2* resulted in a minor deposition of anthocyanin and a slight loss of water in the peel at the injection site. In contrast, when *ERF17* was individually transformed, anthocyanin deposition was evident, and there was no water loss-induced wrinkling of the peel at the injection site. When *ERF2* and *bHLH2* were cotransformed, there was also minor anthocyanin deposition and significant water loss-induced wrinkling of the peel at the injection site, whereas when *ERF17* and *bHLH2* were cotransformed, there was significant anthocyanin deposition but no water loss-induced wrinkling (Figure 8A). The trends of the L* and a*/b* values in the injection regions shown in Figure 8B,C were consistent with the phenotypic results.

RT-qPCR was then used to analyze the expression of *ERF2, ERF17, bHLH2,* and calcium regulatory genes (*CML1, CML2, CML3, CML4,* and *CML5*). The expression of *ERF2, ERF17* and *bHLH2* was significantly increased when the apple peels were injected individually or cotransformed with *bHLH2* (*p* < 0.01, Figure 8D–F). Interestingly, *bHLH2* expression progressively increased with individual transformation of *ERF2, ERF17,* and *bHLH2* or when cotransformed with *bHLH2*. The expression of *CML1, CML2, CML3,* and *CML4* increased significantly (*p* < 0.01) when *ERF2* was individually transformed. There was a slight increase in *CML1, CML2, CML3,* and *CML4* expression when *bHLH2* was individually transformed. The expression of *CML1, CML2, CML3,* and *CML4* decreased when *ERF2, ERF17,* and *bHLH2* were cotransformed (Figure 8G–J). The expression of *CML5* increased significantly (*p* < 0.01) when *bHLH2* was individually transformed and with *ERF2* cotransformation, and the expression of *CML5* was slightly increased when *ERF2* was individually transformed (Figure 8K). In addition, the expression level of *CML5* were significantly higher than those of *CML1, CML2, CML3,* and *CML4* when *ERF2* and *bHLH2* were cotransformed Thus, these results demonstrated that individual transformation of *bHLH2* or cotransformation with *ERF2* significantly activates *CML5* transcription, suggesting that *ERF2* and *bHLH2* cooperate to activate *CML5* to regulate calcium signaling and postharvest apple storage quality.

Promoter cis-acting elements were first predicted for *CML5, ERF2*/*17,* and *bHLH2* and the results are shown in Figure 8L. The 2 kb region upstream of the *CML5* promoter contains one or more cis-acting elements of the DRE core/DRE1 of AP2/ERFs and the G-Box of bHLHs. Then, a dual-luciferase reporter system was used to verify the mode of regulation between *ERF2*/*17* and *bHLH2* and *CML5*. The results showed that *ERF2* and *bHLH2* co-injection significantly activated *CML5* transactivation (Figure 8M). From those it can be concluded that *ERF2*-*bHLH2* coregulation promotes *CML5* expression in apple peels, thereby regulating calcium signaling and postharvest storage quality.

## 3. Discussion

### 3.1. Calcium Deficiency Increases ROS Generation, Reduces H_2_S Production and Postharvest Storage Quality in Apple Peels

Calcium is one of the essential elements for plant growth, and it has important functions in maintaining plant cell structure, resistance to stress and signal transduction [1,2,3,4]. In the present study, the peel of calcium-deficient apples became rough during postharvest storage due to water loss, and they developed distinct bitter pit compared to calcium-sufficient apples (Figure 1A,B). These phenotypic changes were consistent with those reported in previous studies [6,9,10]. In addition, compared to calcium-sufficient peels, calcium-deficient peels produced less H_2_S (Figure 1C–E), significantly increased O_2_^•−^, MDA, total phenol and flavonoid contents, and PPO activity, and decreased POD activity (Figure 2). The increase in ROS and antioxidants such as total phenol and flavonoid in calcium-deficient peels may be caused by bitter pit disorder due to calcium deficiency, which was also consistent with previous reports [5,6,7]. The result of PCA shows that calcium content, ROS, and H_2_S production were main factors between calcium-deficient and calcium-sufficient apple peels. The above results indicated that calcium deficiency in apples reduced H_2_S production and increased ROS production, thereby reducing postharvest fruit storage quality. These results are in agreement with those reported previously [38]. However, the specific regulatory network that exists between calcium signaling and the production of H_2_S and ROS is currently unknown and rarely reported, and further research is needed.

### 3.2. The ERF2-bHLH2 Coactivate the CML5 Expression and Coregulate Downstream Responses in Calcium-Deficient Apple Peels

Calmodulin-like (CML) protein in plant tissues is an important calcium receptor protein in the process of signal transmission. In the present study, the expression of *CML5* was negatively correlated with ROS contents, but positively correlated with POD activity, flavonoids, total phenols, and calcium content (Figure 7), suggesting the potential importance of *CML5* in regulating ROS metabolism. There are numerous studies reporting that CMLs are involved in regulating a variety of abiotic stress processes. For example, *SlCML37* enhances cold tolerance in tomato fruit [39]; *CmCML13* improves drought resistance in *Arabidopsis* [40]; *CML21* is involved in abiotic stress response in grapevine [41]. Thus, it can be hypothesized that *CML5* may be involved in regulating postharvest storage quality and antioxidant-related processes in calcium-deficient apple peels. 

Furthermore, the expression levels of *ERF2*, *ERF17,* and *bHLH2* were screened based on their significant positive correlation with *CML5* and they were significantly positively correlated with the H_2_S production related genes (*DCD1, DCD2, DCD3, DCD4, LCD1, LCD2, LCD3, SiR1,* and *OASTL1*). The results of the apple transient transformation experiment and the dual-luciferase reporter system experiment showed that the co-transformation of *ERF2* and *bHLH2* significantly activated the transcription of *CML5* (Figure 8). Thus, the *ERF2*-*bHLH2*-*CML5* module may be not only involved in the regulation of calcium signaling, but also in the regulation of H_2_S production in postharvest calcium-deficient apples. It was reported that H_2_S could reduce ROS production and increase antioxidant capacity, thereby extending postharvest life of banana [42] and inhibiting enzymatic browning of fresh-cut Chinese water chestnuts [43]. Meanwhile, in *Arabidopsis thaliana* roots, H_2_S induces ROS accumulation, thus inducing the appearance of Ca^2+^ signal [44,45]. In the present study, calcium deficiency caused an increase in ROS production, a decrease in H_2_S production in apple peels and a reduction in post-harvest fruit quality, while further research was needed on how H_2_S was involved in calcium regulation and affected post-harvest fruit quality. Recent studies have also shown that H_2_S emission induced by chromium (Cr^6+^) stress could be modulated by the Ca^2+^ level [46]. However, the molecular mechanism of Ca^2+^-induced endogenous H_2_S emission has rarely been reported. Therefore, the mechanism of how the *ERF2*-*bHLH2*-*CML5* module regulates calcium signaling and H_2_S production in calcium-deficient apples, leading to reduced quality in postharvest storage apples, needs further investigation.

The anthocyanin content of calcium-deficient peels did not differ significantly from calcium-sufficient peels at 0 and 7 DAS, but was significantly lower at 14 and 21 DAS (Figure 2C), indicating that calcium deficiency reduced anthocyanin synthesis in postharvest stored apple peels. Moreover, in the apple transient expression assays experiment, anthocyanin deposition was observed in the peel at the injection site when *ERF2, ERF17,* and *bHLH2* were individually transformed or cotransformed (Figure 8A), suggesting that *ERF2*, *ERF17,* and *bHLH2* may not only be involved in calcium regulation in postharvest stored apples, but also in regulating the anthocyanin biosynthesis pathway in the peel of postharvest stored apples. The mechanism of *ERF2*, *ERF17,* and *bHLH2* in regulating anthocyanin biosynthesis still needs further investigation. 

## 4. Conclusions

In summary, it can be concluded that the production of ROS, total phenols and flavonoids was increased in calcium-deficient peels compared to calcium-sufficient peels, but calcium content, H_2_S production, anthocyanin content, and POD activity were reduced. Calcium content, ROS, and H_2_S production were main factors between calcium-deficient and calcium-sufficient apple peels by PCA analysis. Four CMLs, seven AP2/ERFs, and three bHLHs were screened from transcriptome data analysis. In addition, *ERF2*-*bHLH2* co-activated *CML5* transcription and *ERF2, bHLH2,* and *CML5* expression levels were significantly positively correlated with H_2_S production genes. Thus, the *ERF2*-*bHLH2*-*CML5* module is not only involved in the regulation of calcium signaling, but also in the regulation of postharvest quality of fruit and H_2_S production under calcium deficiency in apples. These findings improved the understanding of the molecular basis of postharvest quality decline in calcium-deficient fruit, and the relationship between calcium and endogenous H_2_S.

## 5. Materials and Methods

### 5.1. Plant Materials and Treatment

The calcium-sufficient and calcium-deficient ‘Honeycrisp’ apples used in this study were provided by the Shandong Academy of Agricultural Sciences (Tai’an, China). Postharvest calcium-sufficient and calcium-deficient apples of similar size and free from pathogen infection were selected for sealed storage in glass containers. In total, 20 calcium-sufficient apples and 20 calcium-deficient apples were placed in individual sealed containers, fumigated with distilled water to maintain a relative humidity of approximately 85% and stored at 25 °C for treatment. After 0, 7, 14, and 21 days after storage (DAS), five calcium-sufficient and calcium-deficient apples were randomly selected for peel sampling. Immediately after stripping, the peels were chilled in liquid nitrogen and stored at −80 °C. Some samples were utilized for the determination of physiological parameters, and some samples were utilized for RNA extraction. Three biological replicates were prepared for all samples. In addition, the ‘Honeycrisp’ apples at 60 days after full blooming used in this study were provided by Shandong Academy of Agricultural Sciences (Tai’an, Shandong Province, China) for transient transformation experiments, and were observed 5 days after injection. Apple peels from the injection sites were obtained to measure the related gene expression levels.

### 5.2. Determination of Calcium Content

A sample of freeze-dried peel (1 g) was dissolved in 1 mol·L^−1^ HNO_3_. Three replicates of each sample were examined. The samples were then diluted with a 5% solution of LaCl_3_. The calcium content of the apple peel samples was measured by atomic absorption spectrometer (Hitachi Z2000, Tokyo, Japan) with an air–acetylene flame [47]. The final calcium content in the peel was expressed as milligrams per kilogram dry weight.

### 5.3. Determination of H_2_S Production in Apple Peels

Lead sulfide method [48] was used to measure H_2_S production capacity. In the presence of excess substrate Cys and cofactor pyridoxal-5′-phosphate (PLP), a specific reaction between H_2_S and lead acetate was used to form a black precipitate (lead sulfide) which can be captured and observed on filter paper containing lead acetate. A sample of fresh apple peel (1 g) was dissolved in PBS supplemented with 10 mM Cys and 10 mM PLP. Six lead acetate H_2_S detection papers (Sigma, Darmstadt, Germany) were placed above the liquid phase in a closed container (covered 96-well plate) and incubated 2–5 h at 37 °C until lead sulfide darkening of the paper occurred.

The changes in H_2_S production capacity were assayed according to Hu’s method [49]. In brief, a lead acetate H_2_S detection paper color was assayed by a colorimeter (model WSC-100, Konica Minolta, Tokyo, Japan) of each paper, and the values of L*, a*, and b* were obtained by averaging the data of three sites.

### 5.4. Determination of Total Phenol, Flavonoid, and Anthocyanin Contents

The method of Piri and Mullins [50] was used to determine the total phenol content of apple peels using a spectrophotometer at 280 nm. The calibration curve was generated using gallic acid as a reference standard.

By measuring the absorbance at 510 nm, the content of flavonoids was determined by aluminum chloride colorimetry [51]. Rutin was applied as a calibration standard. 

The method proposed by Lee and Wicker [52] was used to obtain and measure the anthocyanin content. Apple peels (2 g) were ground with 10 mL of 1% hydrochloric acid methanol solution. The absorptions were measured at 530, 620, and 650 nm, and the anthocyanin content is presented as milligrams per gram fresh weight.

### 5.5. Assay of ROS (Superoxide Anion and Hydrogen Peroxide) and Malondialdehyde (MDA)

Superoxide anion (O_2_^•−^), hydrogen peroxide (H_2_O_2_), and malondialdehyde (MDA) production were determined based on methods previously published by Hu [50]. The MDA content of apple peels was measured using the thiobarbituric acid (TBA) reaction with values expressed as μmol g^−1^. The content of H_2_O_2_ and the production of O_2_^•−^ were presented as μmol g^−1^ and nmol g^−1^ min^−1^, respectively.

### 5.6. Determination of POD, PPO Activity, and Soluble Protein Content

Guaiacol peroxidase (POD) activity was determined based on our previously published method [49] with some modifications. Apple peels (2 g) were homogenized in 2.5 mL of extraction buffer and centrifuged at 13,400× *g* for 30 min at 4 °C. The supernatant obtained was used to measure the activity of POD.

The methods of Benjamin and Montgomery [53] and Beaudoin-Eagan and Thorpe [54] were used to measure the activity of polyphenol oxidase (PPO). Apple peels (2 g) were used for the preparation of PPO enzyme by homogenization in 2 mL of sodium phosphate buffer (50 mM, pH 6.8). POD and PPO activities were expressed as active unit (U) per milligram of protein, and 1 U means 1 μmol product per min. 

Bradford’s [55] method was applied to measure the soluble protein content, and the absorbance value of soluble protein was recorded at 595 nm.

### 5.7. Transcriptome Data Analysis

Transcriptome data of calcium sufficient and calcium deficient apple peels were published in the NCBI database (https://www.ncbi.nlm.nih.gov/bioproject/PRJNA733599, accessed on 15 September 2021) by Qiu et al. [56]. The three sequenced conditions in this transcriptome were T01, diseased peel of calcium-deficient apples; T02, healthy peel of calcium-deficient apples; and T03, peel of calcium-sufficient apples, and each condition had two replicates. The sample peels used for transcriptome were taken from ripe apples. Differentially expressed genes (DEGs) were screened from this transcriptome data (the screening criteria for DEGs are generally: Fold Change ≥ 2 and FDR < 0.01.). 

Gene Ontology (GO) is an officially standardized international classification system for the functional classification of genes, that describes the attributes of genes and their outputs in any organism. Kyoto Encyclopedia of Genes and Genomes (KEGG) analysis provided information and further understanding of secondary metabolites induced by calcium signaling. Then, DEGs were subjected to GO classification and KEGG enrichment analysis.

### 5.8. RNA Extraction and Real-Time Quantitative PCR Analysis

Total RNA from diseased peels of calcium-deficient apples and calcium-sufficient apple peels at 0, 14, and 21 DAS as well as transiently infected apple peel samples was extracted using the Plant RNA Isolation Kit (Foregene, Chengdu, China) and first strand ribonucleic acid was synthesized using a Primer Reverse Transcription Master Mix Kit (Takara, Tokyo, Japan). The RT-qPCR primers are shown in Table S1. The expression levels of *MdTUB* (*TUB*, accession number GO562615) and *MdUBQ* (*UBQ*, accession number MDU74358) were used as normalization genes, and the expression of relative genes was determined with the 2^−ΔΔCT^ method. Three biological replicates were used for all analyses.

### 5.9. Prediction of Cis-Acting Elements in Gene Promoters

The upstream 2 kb promoter sequence of the target gene was taken from the NCBI (https://www.ncbi.nlm.nih.gov/, accessed on 24 September 2021), and the sequence was then input into the PlantPAN 3.0 (http://plantpan.itps.ncku.edu.tw/index.html, accessed on 24 September 2021) for cis-acting element prediction.

### 5.10. Transient Expression Assays in Apple Peels

In the transient transformation experiments, PCR amplification was performed using Phanta^®^ Super-Fidelity DNA Polymerase (Vazyme, Nanjing, China), and the full-length coding sequences of *ERF2*, *ERF17,* and *bHLH2* were introduced into the pSAK277 vector using *EcoRI* and *XbaI* with the 35S promoter as a control. The primer sequences are listed in Table S2. The integration constructs were chemically transformed into the *Agrobacterium tumefaciens* GV3101 strain, and the cells were cultivated at 28 °C for 2 d. The detailed approach for the infiltration experiment has been previously reported by Voinnet et al. [57]. Five apples of similar size and growth were selected for infiltration in each combination, with four sites infiltrated in each apple. At 5 days after infiltration, apple peels were collected for RNA extraction. The negative controls were empty vector infiltrations (pSAK277).

### 5.11. Dual-Luciferase Reporter Assay of Tobacco Leaves

To construct the dual-luciferase reporter vector, the 2 kb upstream promoter region of *CML5* (from the ATG start codon) was amplified from the genomic DNA of peels from ‘Honeycrisp’ apples and inserted into a pGreen II 0800-LUC binary vector. In addition, the injection solutions were prepared as in Section 5.10. *Agrobacterium* cells harboring the pGreen II 0800-LUC recombinant vector and pSAK277 vector, *ERF2*/*17*, *bHLH2* were mixed at a 1:9 ratio. The mixture of *Agrobacterium* cells was injected into young *N. tabacum* leaves that were 2 weeks old. At 48-72 h after infiltration, the LUC and Ren activity were measured with an E1910 Dual-Luciferase^®^ Reporter Assay System (Promega, Madison, WI, USA).

### 5.12. Statistical Analysis

For the statistical analyses, Student’s *t*-test and one-way ANOVA were performed. Significance was indicated by asterisks * (*p*< 0.05) or ** (*p*< 0.01) or different letters. The main factors were assessed by reducing dimensionality when conducting the PCA. R studio software was used for correlation analysis and heatmap analysis. All samples were assessed at least three times independently, and all data are represented as the mean ± SD.

## Figures and Tables

**Table 1:** The factors score of all the metabolites by principal component analysis in apple peels.

Component Name	PC1 (84.7%)	PC2 (15.3%)
H_2_O_2_ content	9.97 × 10^−1^	−8.25 × 10^−2^
O_2_^•−^ production rate	8.25 × 10^−2^	9.97 × 10^−1^
Calcium content	2.01 × 10^−4^	−2.49 × 10^−4^
Ratio of a*/b*	1.99 × 10^−4^	−1.64 × 10^−4^
Soluble protein content	9.53 × 10^−5^	−7.17 × 10^−5^
Flavonoid content	6.48 × 10^−5^	3.87 × 10^−4^
Total phenolics content	4.70 × 10^−5^	1.37 × 10^−4^
Anthocyanins content	1.46 × 10^−5^	−1.74 × 10^−4^
MDA content	5.56 × 10^−8^	1.86 × 10^−5^
PPO activity	−5.74 × 10^−5^	−1.19 × 10^−4^
POD activity	−5.61 × 10^−4^	8.97 × 10^−4^
L* value	−8.94 × 10^−3^	9.04 × 10^−3^

## Supplementary Materials

The following are available online at https://www.mdpi.com/article/10.3390/ijms222313013/s1.

## Abbreviations

Calcium (Ca/Ca^2+^); Reactive Oxygen Species (ROS); Hydrogen sulfide (H_2_S); Cysteine desulfhydrases (CDs); L/D-cysteine desulfhydrase (LCD/DCD); O-acetyl-L-serine (thiol) lyase (OASTL); sulfite reductase (SiR); Superoxide Anion (O_2_^•−^); Malondialdehyde (MDA); Hydrogen Peroxide (H_2_O_2_); Calmodulin (CaM); Calmodulin-Like Proteins (CML); APETALA2/ethylene-responsive factors (AP2/ERFs); Basic Helix-Loop-Helix (bHLH); Pathogen-Related Proteins (PRs); Days After Storage (DAS); Thiobarbituric Acid (TBA); Guaiacol Peroxidase (POD); Polyphenol Oxidase (PPO); Principal Component Analysis (PCA); Differentially Expressed Gene (DEG); Gene Ontology (GO); WEGO (Web Gene Ontology Annotation Plot); Kyoto Encyclopedia of Genes and Genomes (KEGG); Reads Per Kilobase per Million mapped reads (RPKM); Real Time Quantitative PCR (RT-qPCR); Transcription factors (TFs); Pyridoxal-5′-phosphate (PLP).
