# Supplementary material for: Transcriptomics Reveals the ERF2-bHLH2-CML5 Module Responses to H2S and ROS in Postharvest Calcium Deficiency Apples
Source: Int J Mol Sci. 2021 Dec 1;22(23):13013. doi: 10.3390/ijms222313013 (PMC8657956; doi:10.3390/ijms222313013)
Supplement: Supplementary file 1 [file ijms-22-13013-s001.zip › ijms-1459317-supplementary.pdf]

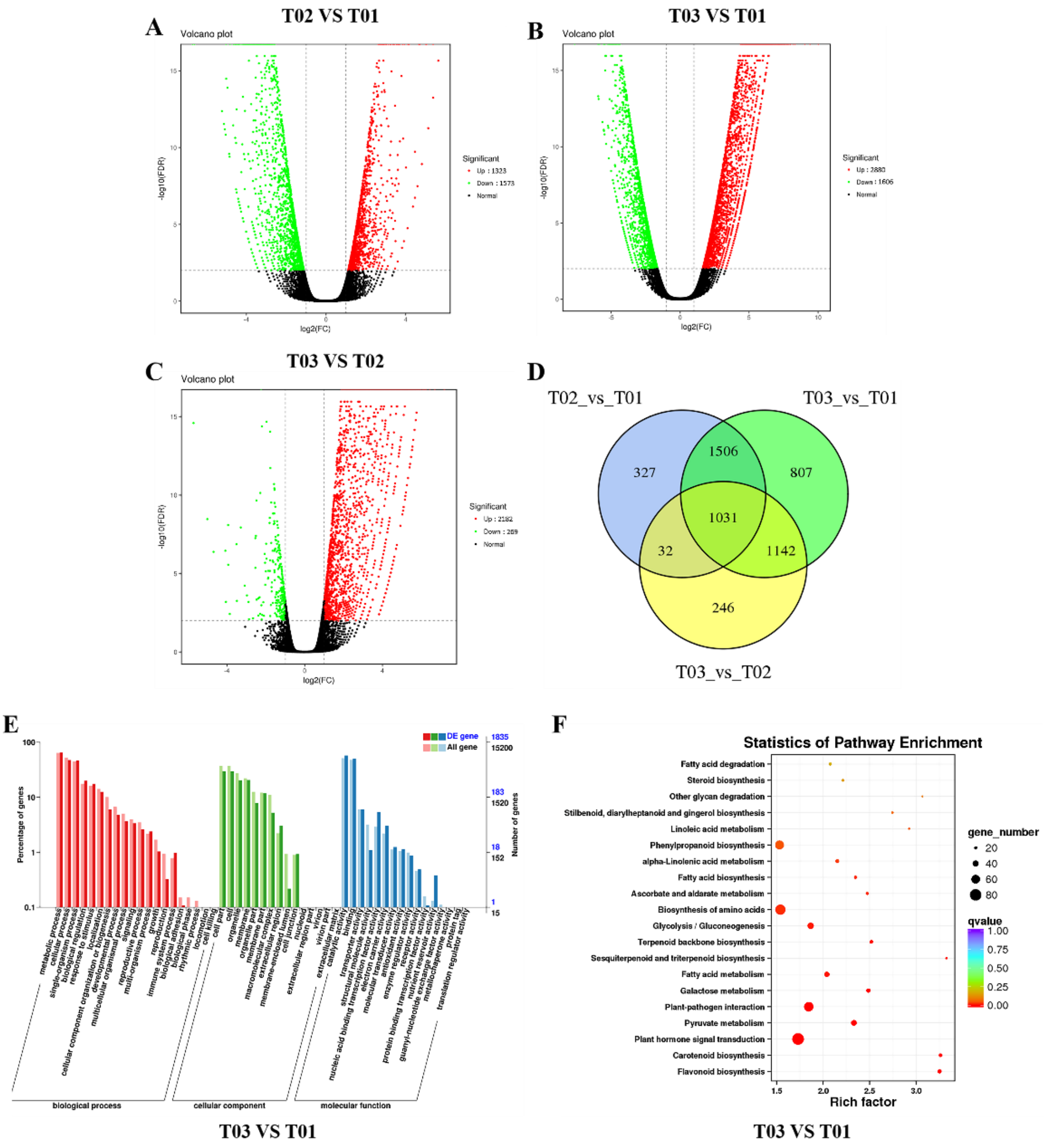

Figure S1. Differential expression analysis and enrichment analysis among sequencing samples. **(A)** Volcano map of DGEs between T02 and T01. **(B)** Volcano map of DGEs between T03 and T01. **(C)** Volcano map of DGEs between T03 and T02. **(D)** Venn diagram of co-DGEs among the samples. **(E)** GO classification and the enrichment analysis of DEGs. **(F)** KEGG enrichment analysis of DEGs. T01, diseased peels of calcium-deficient apples; T02, healthy peels of calcium-deficient apples; T03, peels of calcium-sufficient apples. Negative $\log_{10}$ P values indicate he y-axes, and $\log_2$-fold changes indicate the x-axes.

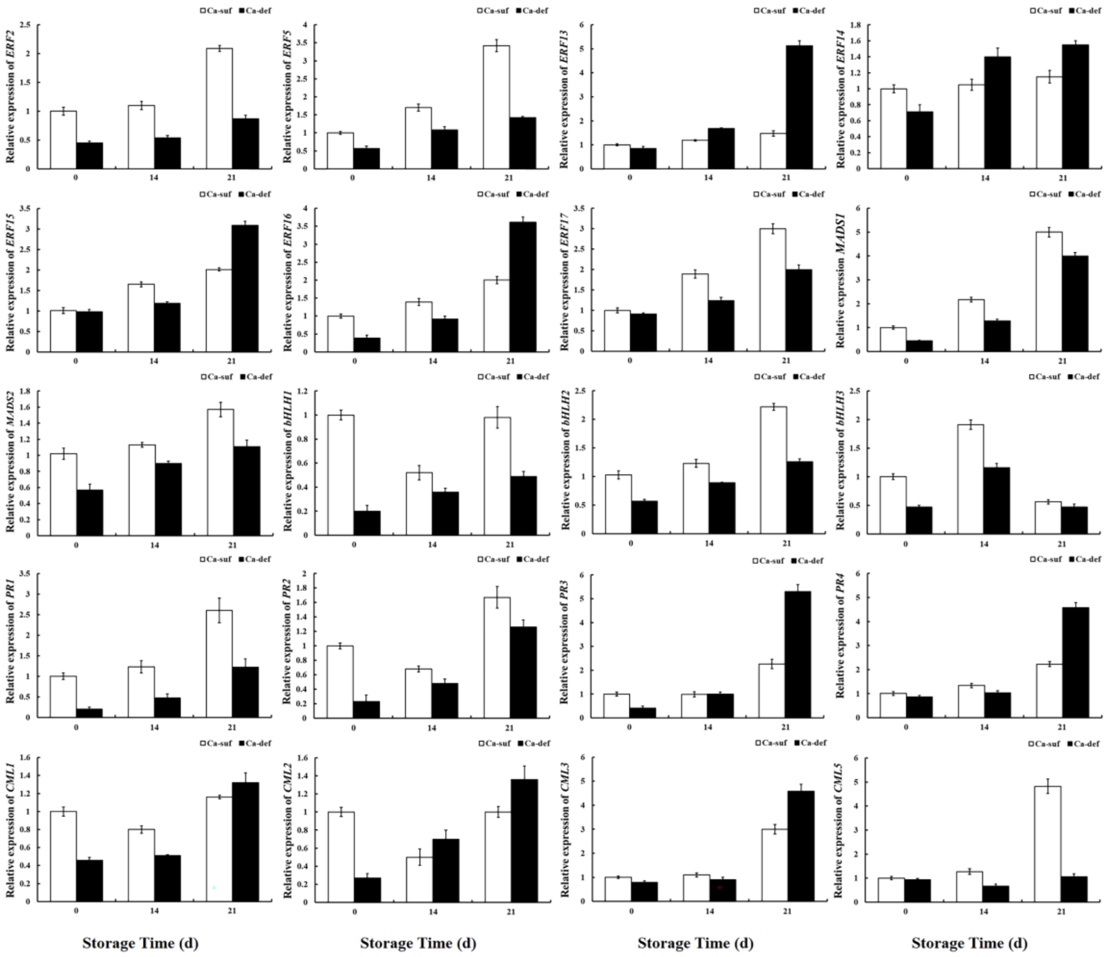

Figure S2. Expression pattern analysis of DEGs (*ERF2, ERF5, ERF13, ERF14, ERF15, ERF16, ERF17, MADS1, MADS2, bHLH1, bHLH2, bHLH3, PR1, PR2, PR3, PR4, CML1, CML2, CML3* and *CML5*) in apple peels under calcium sufficiency and deficiency with storage at 0, 14, 21 DAS. Ca-suf, Ca sufficiency; Ca-def, Ca deficiency. Data are presented as means ± SD (n = 3). Gene expression of *MdUBQ* (*UBQ*, accession number MDU74358) was used as a normalization gene, and relative gene expression was determined using the $2^{-\Delta\Delta CT}$ method to calculate.

Figure S3

Gene name	LOG2 fold change			RT-qPCR		
	Ca-suf	Ca-def	Ca-suf/Ca-def	Ca-suf	Ca-def	Ca-suf/Ca-def
ERF2	628.97	143.02	4.40	4.56	2.27	2.01
ERF5	121.28	9.72	12.48	4.05	2.66	1.52
ERF13	0.54	60.54	0.01	1.88	2.87	0.66
ERF14	6.77	75.66	0.09	3.27	3.81	0.86
ERF15	1.66	71.69	0.02	2.48	4.32	0.57
ERF16	4.24	398.90	0.01	0.96	1.24	0.77
ERF17	177.42	12.83	13.83	4.24	2.46	1.72
MADS1	270.98	86.53	3.13	10.39	9.08	1.14
MADS2	228.16	59.12	3.86	10.85	6.58	1.65
bHLH1	249.49	25.61	9.74	0.62	0.29	2.14
bHLH2	658.29	25.99	25.33	7.11	4.53	1.57
bHLH3	781.14	37.29	20.95	0.22	0.11	2.00
PR1	197.82	88.19	2.24	2.70	1.32	2.05
PR2	21.90	102.37	0.21	1.75	1.34	1.31
PR3	10.39	401.69	0.03	2.66	5.79	0.46
PR4	17.37	3935.32	0.00	8.82	13.36	0.66
CML1	6.33	108.69	0.06	15.03	20.82	0.72
CML2	15.58	201.79	0.08	1.01	1.38	0.73
CML3	10.74	252.54	0.04	5.46	7.82	0.70
CML5	108.54	27.91	3.89	8.16	6.92	1.18

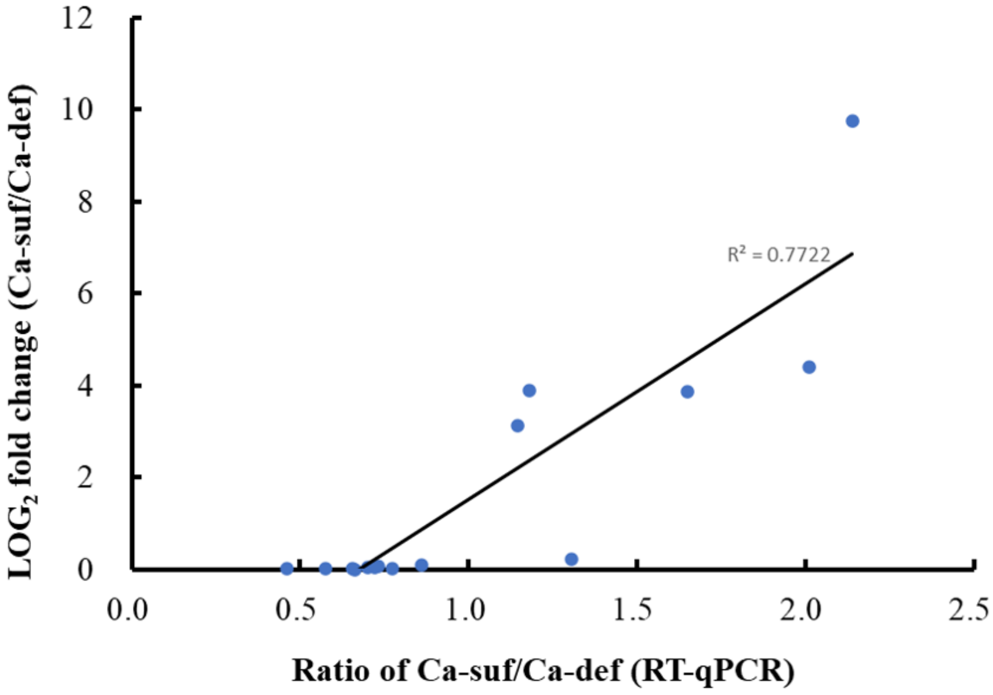

Figure S3. Correlation analysis of the RNA-seq results and RT-qPCR results for the DEGs.

Table S1. Real-time qPCR primers used in this study.

Gene name	Gene ID	Primer sequence (5'-3')
MdERF2	MD13G1163300	F-GTTGTTGTTCGCTGGTGATG<br>R- CCTTCCCTCATAATGACGATCC
MdERF5	MD17G1226700	F- GCCGGTTCTTGCTTCATTTC<br>R- CCCACAGATGAGCTTCGTATC
MdERF13	MD14G1147100	F-GGTTGAATCGGAGGAGAAAGAG<br>R- CACCTGAGCTAGAGCTGAAAC
MdERF14	MD16G1140800	F-GAAGCATCAGAGGAGAAAGAGAG<br>R- CTTGAGTGAGAGCAGAAACCA
MdERF15	MD07G1248400	F-CAGCTACTGCATCTCCTACTTG

		R- GTCCTCCCACCTGATACTGATA
MdERF16	MD06G1130400	F- CCGAAAGAGTTCAGGGAAGAAT
		R- GTGGTGGTCCGTAATACTTTGA
MdERF17	MD01G1177200	F- CGAGTACTCATCCCTCTCTGAA
		R- CGGGTTTCGTTTCGGAATTTG
MdbHLH1	MD13G1150900	F- TCTGATTTCGAATCCCGTAGC
		R- GGTGGAGGTGGTCTTGTTT
MdbHLH2	MD06G1133200	F- CTCTCGGTGAAACTTGACTCTT
		R- GTGACGTGGGACTGAACATAA
MdbHLH3	MD17G1049300	F- CATGTCTAGCAGAGGGTCAAG
		R- CTCAGGAACCAGTTGACGTAAT
MdMADS1	MD06G1204300	F- AGAACCCTGCATGTGCTTATAG
		R- TGAGACAACCTACGCTGAAAC
MdMADS2	MD16G1058500	F- GGCACGGACCAAGTACATAATA
		R- GAGATTTACTACACCAGGCTTCTC
MdPR1	MD10G1017300	F- ACCGTTCCTATATGACTGGAGA
		R-AGAGAGAGAGAGAGAGACCTTTG
MdPR2	MD10G1286300	F- CACATCCATCGTCAACCAAATC
		R- TGCAGAGTCACGGATTTCAG
MdPR3	MD05G1017400	F- ATGGCCTCACCAAGAGTTAAG
		R- GTTGACAGCACCATAGTTAGGA
MdPR4	MD05G1110000	F- GACTCATGCCCAAGACACA
		R- CTATATTGTCCTCCCACGTCAAG
MdCML1	MD12G1087300	F- CGTCATCCTCTTCCTCATACAC
		R- GACCACTCACCCGATCTTTC
MdCML2	MD06G1231000	F- CAAAGACGCCTTCGATCTCTAC
		R- ATCGTGCAGGCCATGTT
MdCML3	MD04G1178900	F- CGCCGCATCTTTGATATGTTC
		R- CAGTGTCTAACCCGAGAAGTG
MdCML4	MD16G1169000	F- GTTCCTCTGGTGGATGGATTC
		R- CTCGTTCCATCCGTGCATAA
MdCML5	MD08G1043100	F- ACGACGGATCGATTCACAAG
		R- AGCACGACAACTCCAATCTC
MdDCD1	MD14G1242800	F- CTGTGACGACCCTGATTACTTC
		R- CCCAGACCTTTGGCATTCT
MdDCD2	MD06G1235700	F- ACACCAAGAACAGCTACGATAC
		R- GGTCATCACGTTGTCCAAGA
MdDCD3	MD06G1235800	F- ATCTCCTGGTTGAGCGTTTAG
		R- CTCAGAGGTGGTAACTGCATAG
MdDCD4	MD13G1105700	F- CTCCAACATTACCAACCCATCT
		R- TCCAACTTTCGCGCCTTATTA
MdLCD1	MD12G1200100	F- ACCGCTCTTCTCTCTCTCTT
		R- CCATTTGGCGATTAGGGTTTG
MdLCD2	MD04G1186700	F- GTTGCACCATCCTGTTGTTTC
		R- TTGCGGGCTATAATCCCTATTC
MdLCD3	MD15G1421300	F- CATCTCGCCGTCTACTTCTTG
		R- GTCCTTACGCTTCCTTCAGTT
MdSiR1	MD05G1081500	F- TCCTCGTCTCTCTCTCAATTCC
		R- GGGATGGTGACATGGTTGATAG
MdOASTL1	MD06G1007300	F- AGTTCGACAACCCTGCTAATC
		R- AACCGTCCTACACCAGAAATG
MdTUB	GO562615	F- ACACGGGGAGGTAGTGACAA
		R- CCTCCAATGGATCCTCGTTA
MdUBQ	MDU74358	F- CTCCGTGGTGGTTTTTAAGT
		R- GGAGGCAGAAACAGTACCAT

Table S2. Primers used in this study for developing the constructs.

The purpose	The primers	The sequences of the primer (5'-3')
For construction of re-combinant vector of pSAK277	*ERF2-EcoR*I-F	actagtggatccaaagaattcATGTGTGGTGGTGCTATCATTTCC
	*ERF2-Xba*I-R	tcattaaagcaggactctagaTTAATACAGAAGCTGCCCTTGTTG
	*ERF17-EcoR*I-F	actagtggatccaaagaattcATGGTAATTAGCTTCCCCTTCTGC
	*ERF17-Xba*I-R	tcattaaagcaggactctagaTCAAGCACCTTCTCCTTCCCA
	*bHLH2-EcoR*I-F	actagtggatccaaagaattcATGGCAGCCTTTTCATACCAAA
	*bHLH2-Xba*I-R	tcattaaagcaggactctagaTTAATTGAAAGAACACAAGTTGCTGT
For construction of pGreen II 0800-LUC binary vector	*CML5-Hind*III-F	gtcgacggtatcgataagcttTAAAGGTATGAGCACGGAAACTGA
	*CML5-BamH*I -R	cgctctagaactagtggatccTGCTGTCGTCTGCAACAATTCA
